# Optimization of Preparation Conditions for Side-Emitting Polymer Optical Fibers Using Response Surface Methodology

**DOI:** 10.3390/polym12123062

**Published:** 2020-12-21

**Authors:** Xianjin Hu, Kun Yang, Cheng Zhang

**Affiliations:** 1 School of Textile Science and Engineering, Tiangong University, Tianjin 300387, China; xjhu2020@163.com; 2 School of Electrical Engineering and Automation, Tiangong University, Tianjin 300387, China; zhangcheng@tjpu.edu.cn

**Keywords:** side-emitting polymer optical fibers, solvent treatment method, center composite design, response surface methodology, optimization

## Abstract

Polymer optical fibers (POFs) were used for preparing side-emitting polymer optical fibers (SPOFs), which were processed with acetone and n-hexane combined in selected proportions by a solvent treatment method. The effects of the volume ratio of acetone to n-hexane and treatment time on response variable factors were investigated. The center composite design (CCD) based response surface methodology (RSM), a quadratic model, and a two-factor interaction model were developed to relate the preparation variables of illumination intensity, breaking strength, and rigidity. According to analysis of variance (ANOVA), the factors affecting the optimization of each response factor were determined. The predicted values after process optimization were found to be highly similar to the experimental values. The optimal conditions for the preparation of SPOF were as follows: the volume ratio of acetone to hexane was 1.703, and the treatment time was 2.716 s. The three response variables of SPOF prepared under the optimal conditions were: illumination intensity 19.339 mV, breaking strength 5.707 N, and rigidity 572.013 N·mm^2^.

## 1. Introduction

As a textile material, side-emitting polymer optical fibers (SPOFs) have the advantages of softness, light weight, and flexibility, which can be combined with other fibers to form light-emitting fabric, used in the optical medical field [1]. Thus, the properties and application of SPOFs have gradually become one of the research hotspots in the field of phototherapy [2,3,4,5,6].

Traditionally, SPOFs for commercial use have the disadvantages of having a large diameter and high rigidity, and not being easy to weave and knit [7]. To obtain SPOFs with ideal optical properties, researchers paid more attention to the processing and treatment on their surface structure [8].

There are two main methods for POFs to realize the side-emitting effect [9]: to destroy the fiber structure and to bend the fiber. The former focuses on the change of the POF cladding structure, causing light to leak out of the fiber’s interior. The latter changes the transmission path of light in the core, which cannot satisfy the total reflection condition. Chemical solvent treatment is a method of destroying the cladding structure of the optical fiber, which has the advantages of low cost, convenient operation, and high luminous intensity.

To study the behavior of POFs treated by chemical solvent, a lot of research has been done by some scholars. Merchant et al. [10] applied chemical tapering techniques to prepare POF sensors, which was a significant enhancement of POF devices. They had earlier clarified the treatment of POF with hydrofluoric acid, pure acetone, and organic solvent. Batumaiay et al. [11] studied the reaction of acetone as a chemical solvent treatment agent with POF surface polymer. After a large amount of deionized water rinsing, the white reactant attached to the surface and was sanded with sandpaper to obtain SPOF. Rajan et al. [12] and Bhowmik et al. [13,14] used acetone and methanol to mix. The volume ratio of the mixed solution and the treatment time were determined. The surface morphology and structure of the POFs were characterized. However, the quantitative relationship between the processing factors and the performance of the obtained SPOFs were seldom mentioned.

Based on the above references, acetone and n-hexane were combined in selected proportions for the solvent treatment method. The volume ratio of the mixed solution and treatment time were taken as independent variables, and the illumination intensity, breaking strength, and rigidity were taken as response variables. With the help of CCD [15] based RSM [16,17], the relationship between independent variables and response variables was optimized and analyzed. The optimal preparation parameters were obtained. SPOFs were prepared under optimum conditions, and the predicted values were compared with the actual values. The reliability of the design model was proved by the morphological characterization of SPOFs by scanning electron microscopy (SEM).

## 2. Experimental

### 2.1. Materials

**Figure 1:**
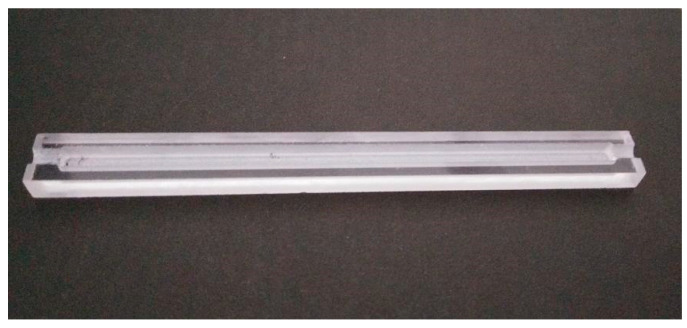
Device for treating POFs.

POFs (Jiangxi Dasheng Plastic Optical Co., Ltd., Jiangxi, China) were used in the experiment. Their core material is polymethyl methacrylate (PMMA), and the cladding material is fluororesin. Characteristics of the POFs are listed in Table 1. Theses fibers were treated in a device that is made with glass and has a groove of 100 mm × 5 mm × 5 mm, as shown in Figure 1. Acetone (99.5%, Tianjin Komeo Chemical Reagent Co., Ltd., Tianjin, China) and n-hexane (99.5%, Tianjin Fengchuan Chemical Reagent Co., Ltd., Tianjin, China) were combined in selected proportions to treat POFs.

### 2.2. Central Composite Design (CCD)

A standard RSM called CCD was used in this investigation. The multivariate quadratic regression equation was used to fit the functional relationship between the factors and response values. By analyzing the regression equations, the optimal process parameters were sought to solve the multivariate problem [18]. CCD naturally divides the factor into two subsets: the first subset estimates linear and two-factor interaction effects, and the second subset estimates the curvature effect. CCD is also characterized by its effectiveness and flexibility. It provides a lot of information about experimental variables and experimental errors with a minimum of test cycles, and its design type can be applied to the operability region and region of interest [19]. In simple terms, CCD can be applied to the fitting of quadric surfaces. Generally, CCD consists of 2^n^ factorial runs with 2n axial runs and n_c_ center runs.

In this study, the independent variables X_1_ and X_2_ were volume ratio and treatment time, respectively. For each categorical variable, a CCD [20] for the two variables consisting of four factorial points, four axial points, and five replicates at the center points were employed, indicating that altogether 13 experiments were required, as calculated from Equation (1):

(1) $${N\;=\;2}^{n}{\;+\;2n\;+\;n}_{c}{\;=\;2}^{2}\;+\;2\;\times \;2\;+\;5\;=\;13,$$

where N is the total number of experiments required and n is the number of factors.

The independent variables were coded to the (−1, 1) interval where the low levels were coded as −1 and the high levels were coded as +1. The axial points were located at (±α, 0) and (0, ±α) where α is the distance of the axial point from the center and makes the design rotatable. The α value was fixed at 1.414, and the factor level table is shown in Table 2.

### 2.3. Preparation of SPOFs

Several POFs [21] with a length of 400 mm were obtained, which were marked 100 mm ± 5 mm in the middle. The marked part of the optical fiber was placed in the trough of the solvent processor, and acetone and n-hexane were combined in selected proportions as described in Table 2 and were dripped into the trough for the corresponding reaction time. After solvent treatment, the optical fibers were washed by high-pressure water and immersed in an ultrasonic cleaner (KQ-500VDE, Kunshan Ultrasound Instrument Co., Ltd., Kunshan, China) to remove the white adhesion on the fiber’s surface. After the above operation, the SPOFs were prepared.

### 2.4. SEM Text of SPOFs

A Phenom Pure scanning electron microscope (SEM, Phenom Pure, FUNER, Eindhoven, The Netherlands) was used to observe the surface morphology and structure of SPOFs after acetone and n-hexane were combined in selected proportions and with different time treatments.

### 2.5. Illumination Intensity Test

**Figure 2:**
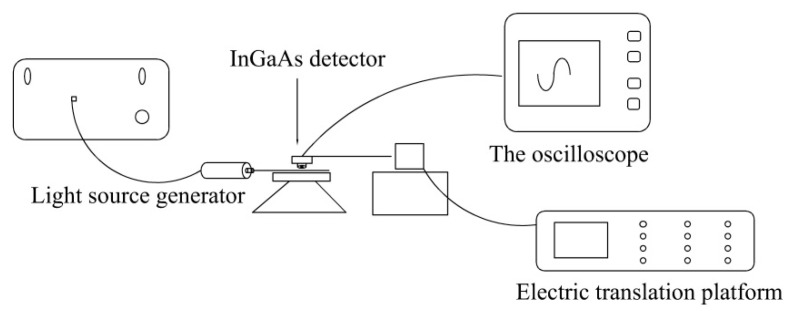
Illumination intensity test system.

An illumination intensity [22] test system, as shown in Figure 2, was built in a darkroom. The system includes a light source generator (LSB-RD-VIS-CW-30, Tianjin junfeng technology co. LTD, Tianjin, China), electric translation platform (42BYG250Bk-B, Beiyang company, Tianjin, China), InGaAs detector (DET10N, Thorlabs, Tianjin, China), and oscilloscope (DS1102D, RIGOL, Tianjin, China). The light source power was 0.6 W. When the light source generator was 150 mm apart from the side luminous region of the SPOFs, the InGaAs detector collected the first data and converted the optical signal into an electrical signal. Then, nine movements with 10 mm intervals were carried out by using the electric translation platform. The luminous data were collected in turn, and the effective values were recorded by the oscilloscope. Six groups of parallel experiments were set up, and the average value was taken as the valid data.

### 2.6. Breaking Strength Test

The breaking strength test equipment was a vertical universal strength tester (5969B10860, INSTRON, New York City, NY, USA). The length of the tested sample between the two clamping devices was 100 mm. The constant tensile speed was 100 mm/min. Six groups of parallel experiments were set up, and the average values of the six groups of parallel experiments were summarized as the final effective data.

### 2.7. Rigidity Test

The samples were clamped on both sides of the universal strength tester (5969B10860, INSTRON, America) in a straight line with a clamping distance of 100 mm. When the fixture distance was reduced to 30 mm, the optical fiber was U-shaped. The bending radius of the optical fiber was measured. Then, the elastic force of the optical fiber on the machine was collected. Finally, the rigidity was calculated by Equation (2). Six groups of parallel experiments were set up, and the average values of the six groups of parallel experiments were summarized as the final effective data.

(2) $${B\;=\;F\pi r}^{2},$$

where B represents the rigidity, F is elasticity at the lowest measured point (N), and r is the measured minimum bending radius of optical fibers (mm).

## 3. Results and Discussion

### 3.1. Model Fitting and Statistical Analysis

Multivariate regression fitting analysis was performed using Minitab17 software, which can perform linear two-factor interaction and quadratic polynomial or high-order model fitting on the data, and is also used to evaluate the statistical significance of the regression equation.

### 3.2. Data Processing

Table 3 shows the complete design matrix of the experiment and inputs of the response variables: illumination intensity (Y_1_), breaking strength (Y_2_), and rigidity (Y_3_). Each response is used to establish an empirical model, which uses the second-degree polynomial equation given by Equation (3) to correlate the response with variables:

(3) $${Y\;=\;\beta }_{0}+\overset{k}{\underset{i=1}{\sum }}\beta_{i}X_{i}\overset{k}{\underset{i=1}{\sum }}\beta_{\mathrm{ii}}X_{i}^{2}+\overset{k}{\underset{i=1}{\sum }}\overset{k}{\underset{j=1}{\sum }}\beta_{\mathrm{ii}}X_{i}X_{j}+\varepsilon ,$$

where Y represents the process response, β_0_ is the free term, k is the number of patterns, i and j are index numbers for patterns, X_i_ …X_k_ are coded independent variables, β_i_ is the first-order effect, β_ii_ is the quadratic effect, β_ij_ is the interaction effect, and ε [23] is the random error accounting for the discrepancies or uncertainties between predicted and observed values.

### 3.3. Application of Central Composite Model

CCD was used to develop correlation between the SPOFs’ preparation variables and the illumination intensity (Y_1_), breaking strength (Y_2_), and rigidity (Y_3_). It was found that the illumination intensity of the SPOFs prepared by the solvent treatment method ranged from 2.670 to 9.238 mV, the breaking strength ranged from 5.133 to 6.396 N, and the rigidity ranged from 496.218 to 726.526 N·mm^2^.

The central composite model (Table 3) can enable the development of mathematical equations from which each response Y = f(X) was estimated as a function of X_1_ and X_2_ and calculated as the sum of a constant, two first-order effects (X_1_, X_2_), one interaction effect (X_1_X_2_), and two second-order effects (X_1_^2^, X_2_^2^). The final model equations obtained in terms of uncoded variables are given in Equations (4)–(6).

According to the sequential model sum of squares, the models were selected based on the highest order polynomials where the additional terms were significant and the models were not aliased. The final empirical models in terms of coded factors after excluding the insignificant terms for illumination intensity (Y_1_), breaking strength (Y_2_), and rigidity (Y_3_) are shown in Equations (4)–(6), respectively:

(4) $$Y_{1}=12.851+6.992X_{1}+2.251X_{2}+{0.613X}_{1}^{2}-{1.400X}_{1}^{2}-{0.680X}_{1}X_{2}$$

(5) $$Y_{2}=5.5442-{0.3505X}_{1}-{0.2046X}_{2}+{0.1636X}_{1}^{2}+{0.0337X}_{2}^{2}-{0.0195X}_{1}X_{2}$$

(6) $$Y_{3}=588.29-{75.33X}_{1}-{43.68X}_{2}+{18.81X}_{1}^{2}-{1.27X}_{2}^{2}-{0.83X}_{1}X_{2}$$

A positive sign in front of the terms indicates a synergistic effect, whereas a negative sign indicates an antagonistic effect. The quality of the model developed was evaluated based on the correlation coefficient value. According to the regression Equations (4)–(6), the single-form coefficient values, the priority between the main influences of the three response factors, is volume ratio (X_1_) > treatment time (X_2_).

From the significance test of the regression coefficient in Table 4, the X_1_ term in model (4) is significant, and the other terms are not significant; in model (5), X_1_, X_2_, and X_1_^2^ are significant, and other items are not significant; in model (6), X_1_ and X_2_ are significant.

### 3.4. Statistical Analysis

ANOVA is a statistical technique that subdivides the total variation in a set of data into component parts associated with specific sources of variation for the purpose of testing hypotheses on the parameters of the model [24,25]. Table 5 summarizes the results of ANOVA to test the reliability of the model. The average square values were calculated by dividing the sum of squares of each variant source by their degrees of freedom, and then using a 95% confidence level (alpha = 0.05) to determine the statistical significance of all of the analyses.

Table 5 shows the regression ANOVA predicting the parameters of the response surface quadratic model Y_1_–Y_3_ processed by synthesis. Results are evaluated using various descriptive statistics, such as *p*-values, *F*-values, and degrees of freedom (df). When *p* < 0.05, the model is significant, and a small probability value (*p* < 0.001) indicates that the model is highly significant and can be used to accurately predict the response function. All linear parameters were significant, and the quadratic parameters were significantly different, except for the stiffness, for which the *p*-value was less than 0.05; the interaction term parameters were not significant, and the *p*-value was greater than 0.05.

The ANOVA results for three parameters (Y1–Y3) showed significant RSM with high R^2^ values, which varied from 0.966 to 0.992 (Table 5). The R^2^ valued representation model took into account the variability of data, while R^2^ (adj) modified the R^2^ valued by considering the number of covariates or predictors in the model. These higher R^2^ coefficients ensured satisfactory adjustment of the quadratic models to the experimental data [26].

R^2^(adj) values of 0.986, 0.979, and 0.941 for the three models Y_1_, Y_2_, and Y_3_, respectively, were also high, indicating the significance of the models [27]. Therefore, the RSM was used to predict the variation in these three parameters within the range of the chosen variables.

### 3.5. Effects of Model Parameters and Their Interactions

The Minitab17 software was used to produce three-dimensional (3D) response surfaces and two-dimensional (2D) contour plots [28,29]. The 3D surface and 2D contour plots are graphical representations of the regression equation for optimizing reaction conditions, which are the most useful methods for revealing reaction system conditions. In these plots, the response functions of two factors are given, while all other factors are at a fixed level.

**Figure 3:**
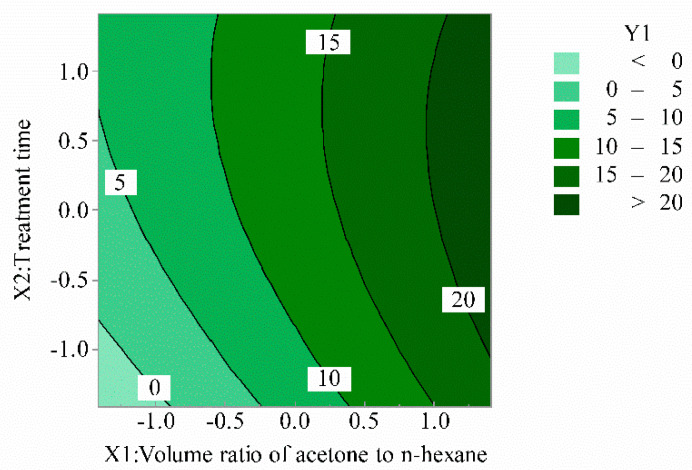
Volume ratio of acetone to n-hexane and treatment time for illumination intensity (2D).

**Figure 4:**
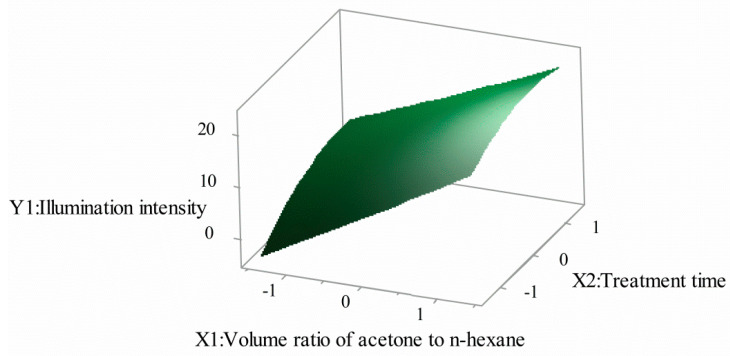
Volume ratio of acetone to n-hexane and treatment time for illumination intensity (3D).

Figure 3 and Figure 4 show the effect of the volume ratio of acetone to n-hexane and the treatment time on the illumination intensity. It can be seen that with the increase of the volume ratio of acetone to n-hexane, the illumination intensity of SPOFs increased. When the volume ratio of acetone to n-hexane was at a certain level, the illumination intensity of SPOFs increased with the increase of the treatment time, and then tended toward a relatively stable state. Increasing the proportion of acetone in the mixed solution increased the polarity of the mixed solution. The greater the polarity of the mixed solution and the longer the treatment time, the greater the corrosion degree of the fluororesin in the POFs’ cortex. The more light that leaked in the transmission of POFs, the higher the side illumination intensity.

**Figure 5:**
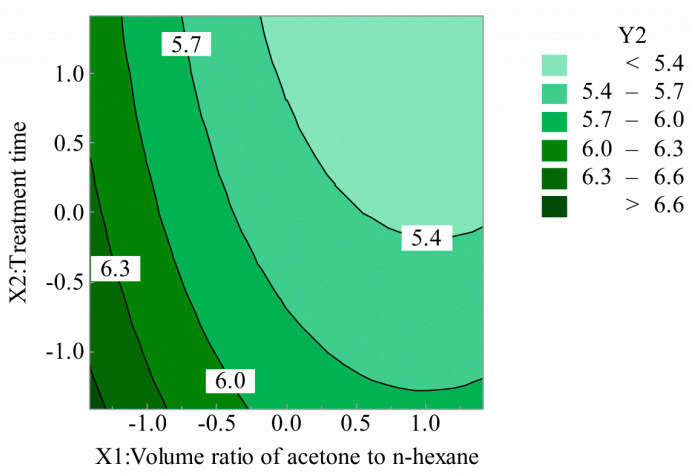
Volume ratio of acetone to n-hexane and treatment time for breaking strength (2D).

**Figure 6:**
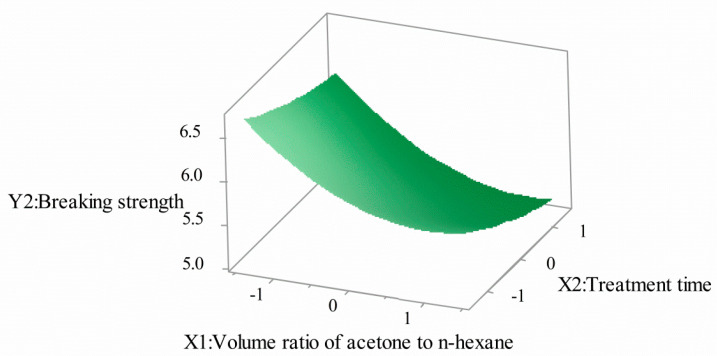
Volume ratio of acetone to n-hexane and treatment time for breaking strength (3D).

Figure 5 and Figure 6 are compositions of the effects of the volume ratio of acetone to n-hexane and treatment time on breaking strength. It can be seen that with the increase of the volume ratio of acetone to n-hexane, the breaking strength decreased rapidly at first and then tended to be flat. With the increase of treatment time, the breaking strength decreased, but the overall decline curve was gentle. When the volume ratio of acetone to n-hexane began to increase, the cladding tissue of POFs slowly expanded, the structure of fluororesin changed, and the physical and mechanical properties decreased. However, with the volume ratio of acetone to n-hexane increasing again, the cladding tissue of optical fiber dissolved and fell off. The treatment time deepened the swelling and dissolution of the cortex.

**Figure 7:**
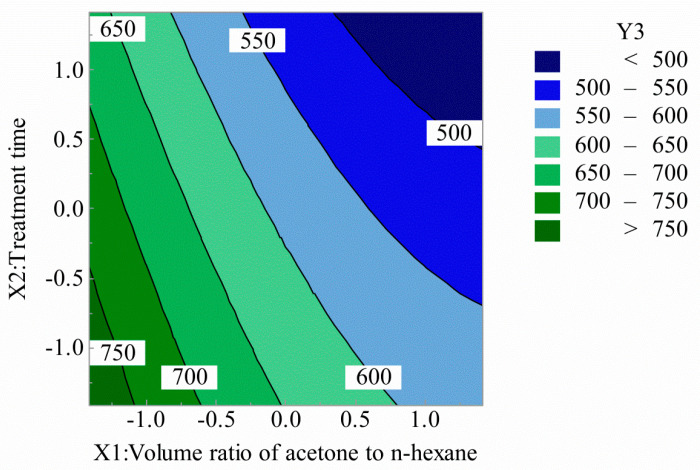
Volume ratio of acetone to n-hexane and treatment time for rigidity (2D).

**Figure 8:**
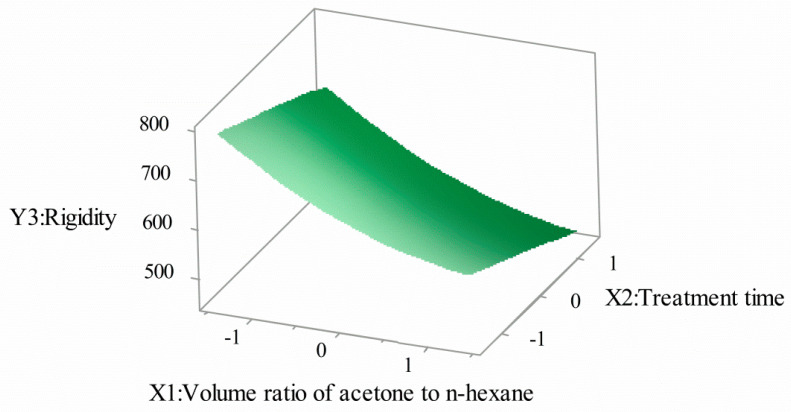
Volume ratio of acetone to n-hexane and treatment time for rigidity (3D).

Figure 7 and Figure 8 show the interaction effect of the acetone volume ratio with the hexane volume ratio and treatment time on rigidity. It can be seen that the rigidity of SPOFs decreased with the increase of the volume ratio of acetone to n-hexane. With the increase of treatment time, the rigidity decreased, but the overall downward trend was gentle. The structure and properties of fluororesin in the optical fiber cortex were changed, the plastic deformation of optical fiber was enhanced, and the rigidity of SPOFs was reduced.

The contour map and response surface map were analyzed by Minitab software. When the volume ratio of acetone to n-hexane was 1.703 and the treatment time was 2.716 s, the illumination intensity and breaking strength could be satisfied, optimizing the maximum value and minimum value of the rigidity optimization. The optimized result was an illumination intensity of 19.339 mV, breaking strength 5.707 N, and rigidity 572.013 N·mm^2^ (Table 6).

### 3.6. Optimal Conditions

To confirm whether the model was sufficient to predict the solvent treatment method for preparing the SPOF process parameters, a new experiment was carried out at the optimum level, as shown in Table 7. The results in Table 5 indicate that there was good agreement between the predictions, and the best level of experimental results gave a high degree of validity of the model.

**Figure 9:**
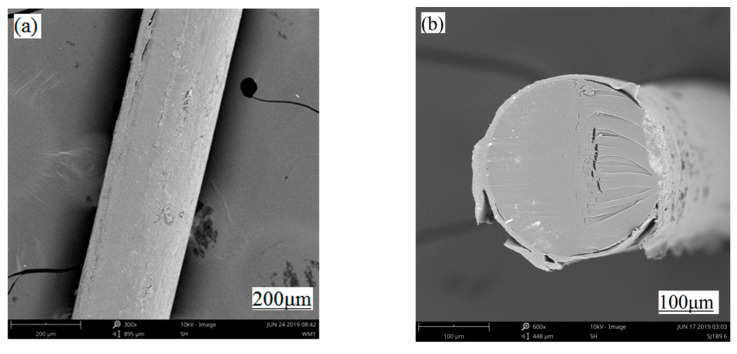
SEM image of the surface and cross-sectional views of side-emitting polymer optical fiber prepared under optimal conditions (**a**) Radial image (**b**) Cross-sectional image.

Figure 9a,b shows the SEM surface and cross-sectional views, respectively, of SPOFs obtained under optimal preparation conditions. It can be seen from Figure 9a,b that the surface of the fiber had a convex surface, a small part of the cladding structure was detached, and the surface was relatively flat. The swell effect was mainly caused by the fiber skin layer as a whole, thereby increasing the inherent loss of the fiber, potentially causing the light to leak from the fiber. The outermost part of the cortex had a dissolution effect, which increased the illumination intensity of the fiber while reducing the rigidity, causing the breaking strength to not attenuate too much. Under optimal preparation conditions, preparing SPOFs using the solvent treatment method ensured not only the luminescent property, but also the physical and mechanical properties. This was an ideal preparation scheme for SPOFs.

## 4. Conclusions

An effective method for preparing SPOFs by solvent treatment was established. The CCD-based RSM was used to evaluate and optimize the effects of the volume ratio of acetone to n-hexane and treatment time on illumination intensity, breaking strength, and rigidity. It was found that in the process of preparing SPOFs by using the solvent treatment method, the fiber cortex structure had two effects of swelling and dissolution, causing the light transmittance and physical and mechanical properties of the fiber to change. The combination of RSM based on CCD proved to be a powerful tool in the optimization of SPOF preparation process parameters. The optimum conditions for preparing SPOFs is with a solvent treatment area volume ratio of acetone to n-hexane of 1.703 and a treatment time of 2.716 s. The results of the three properties of the SPOFs prepared by using the optimized values are highly consistent with the model prediction results.

## Figures and Tables

**Table 1:** Characteristics of the polymer optical fibers (POFs) used in this study.

Project	Parameter
Fiber diameter (mm)	0.250
Fiber cladding thickness (mm)	0.025
Numerical aperture	0.5
Maximum transmission loss (dB/km)	250
Working temperature (℃)	−50–(+70)

**Table 2:** Experimental range and levels of independent variables.

Factor	Range and Levels				
Independent variable	−α	−1	0	1	α
Volume ratio of acetone to n-hexane (X_1_)	0.3	0.5	1	1.5	1.7
Treatment time (X_2_, s)	1.5	4	10	16	18.5

**Table 3:** Full factorial central composite design matrix for two test variables in uncoded units, along with the observed and predicted responses.

Run	Volume Ratio of Acetone to n-hexane, X_1_	Treatment Time, X_2_(s)	Illumination Intensity, Y_1_(mV)	Breaking Strength, Y_2_(N)	Rigidity, Y_3_(N·mm^2^)
1	1(0)	10(0)	7.064	5.481	582.512
2	0.5(−1)	16(1)	4.374	5.876	659.076
3	1.5(1)	4(−1)	6.869	5.584	568.832
4	0.3(−1.414)	10(0)	2.775	6.396	726.526
5	1(0)	10(0)	6.938	5.601	596.322
6	1(0)	10(0)	6.670	5.510	590.384
7	1(0)	18.5(1.414)	7.190	5.355	496.218
8	0.5(−1)	4(−1)	2.670	6.249	715.862
9	1.5(1)	16(1)	7.615	5.133	508.738
10	1(0)	1.5(−1.414)	4.281	5.930	660.651
11	1(0)	10(0)	7.159	5.593	590.010
12	1.7(1.414)	10(0)	9.238	5.409	510.682
13	10(0)	10(0)	7.000	5.536	582.214

**Table 4:** Significance test of coefficients in the regression equation.

Source	Y_1_		Y_2_		Y_3_	
	Coefficient	*p*-Value	Coefficient	*p*-Value	Coefficient	*p*-Value
X_1_	6.922	<0.001	−0.351	<0.001	−75.331	<0.001
X_2_	2.251	<0.001	−0.205	<0.001	−43.683	<0.001
X_1_^2^	0.163	0.568	0.164	<0.001	18.812	0.028
X_2_^2^	−1.400	0.001	0.034	0.128	−1.270	0.857
X_1_X_2_	−0.680	0.101	−0.020	0.474	−0.847	0.929

**Table 5:** ANOVA for the response surface quadratic model.

Source	Degree of Freedom	Sum of Squares	Mean Square	*F*-Value	Prob > *F*
Y_1_ (Illumination intensity, mV)					
Model	5	440.246	88.049	169.832	<0.001
Linear	2	423.909	211.955	408.807	<0.001
Square	2	14.484	7.242	13.972	0.004
Two-Way Interaction	1	1.852	1.852	3.574	0.101
R^2^ = 0.992					
R^2^(adj) = 0.986					
Y_2_ (Breaking strength, N)					
Model	5	1.507	0.301	113.648	<0.001
Linear	2	1.318	0.659	248.487	<0.001
Square	2	0.187	0.094	35.340	<0.001
Two-Way Interaction	1	0.002	0.002	0.573	0.474
R^2^ = 0.988					
R^2^(adj) = 0.979					
Y_3_ (Rigidity, N·mm^2^)					
Model	5	63,218.912	12,643.836	39.346	<0.001
Linear	2	60,655.943	30,327.872	94.382	<0.001
Square	2	2560.324	1280.137	3.982	0.070
Two-Way Interaction	1	2.800	2.800	0.001	0.929
R^2^ = 0.966					
R^2^(adj) = 0.941					

**Table 6:** Multiple response prediction.

Variable	Setting	Response	Fit	SE Fit	95% CI	95% PI
		Y_1_	19.339	0.932	(17.135, 21.542)	(16.554, 22.123)
		Y_2_	5.707	0.067	(5.550, 5.865)	(5.508, 5.907)
		Y_3_	572.013	23.218	(517.132, 626.841)	(502.557, 641.326)
X_1_	1.703					
X_2_	2.716					

**Table 7:** Comparison of the predictive and experimental result optimum values.

Parameter	Optimum Value	Response	Predictive	Experimental
X_1_	1.703	Y_1_	19.339	18.862
X_2_	2.716	Y_2_	5.707	5.736
		Y_3_	572.013	563.647
